# Excessive rest time during active phase is reliably detected in a mouse model of myotonic dystrophy type 1 using home cage monitoring

**DOI:** 10.3389/fnbeh.2023.1130055

**Published:** 2023-03-02

**Authors:** Elisabetta Golini, Mara Rigamonti, Marcello Raspa, Ferdinando Scavizzi, Germana Falcone, Genevieve Gourdon, Silvia Mandillo

**Affiliations:** ^1^Institute of Biochemistry and Cell Biology (IBBC), National Research Council (CNR), Monterotondo, Italy; ^2^Tecniplast S.p.A., Buguggiate, Italy; ^3^Sorbonne Université, INSERM, Institut de Myologie, Centre de Recherche en Myologie, Paris, France

**Keywords:** DVC^®^, DMSXL, DM1, digital biomarker, sleep, excessive daytime sleepiness (EDS), home cage monitoring

## Abstract

Myotonic dystrophy type 1 (DM1) is a dominantly inherited neuromuscular disease caused by the abnormal expansion of CTG-repeats in the 3′-untranslated region of the Dystrophia Myotonica Protein Kinase (DMPK) gene, characterized by multisystemic symptoms including muscle weakness, myotonia, cardio-respiratory problems, hypersomnia, cognitive dysfunction and behavioral abnormalities. Sleep-related disturbances are among the most reported symptoms that negatively affect the quality of life of patients and that are present in early and adult-onset forms of the disease. DMSXL mice carry a mutated human *DMPK* transgene containing >1,000 CTGrepeats, modeling an early onset, severe form of DM1. They exhibit a pathologic neuromuscular phenotype and also synaptic dysfunction resulting in neurological and behavioral deficits similar to those observed in patients. Additionally, they are underweight with a very high mortality within the first month after birth presenting several welfare issues. To specifically explore sleep/rest-related behaviors of this frail DM1 mouse model we used an automated home cage-based system that allows 24/7 monitoring of their activity non-invasively. We tested male and female DMSXL mice and their wild-type (WT) littermates in Digital Ventilated Cages (DVCR) assessing activity and rest parameters on day and night for 5 weeks. We demonstrated that DMSXL mice show reduced activity and regularity disruption index (RDI), higher percentage of zero activity per each hour and longer periods of rest during the active phase compared to WT. This novel rest-related phenotype in DMSXL mice, assessed unobtrusively, could be valuable to further explore mechanisms and potential therapeutic interventions to alleviate the very common symptom of excessive daytime sleepiness in DM1 patients.

## Introduction

Myotonic Dystrophy type 1 (DM1) is a dominantly inherited neuromuscular disorder caused by the expansion of CTG repeats in the *DMPK* gene leading to abnormal RNA processing and alteration of various protein functions (Udd and Krahe, 2012).

Myotonic Dystrophy type 1 is characterized by multi-systemic symptoms including muscle weakness, myotonia, cardio-respiratory problems, and several central nervous system (CNS) related symptoms that increase the disease burden and negatively affect the quality of life of patients (Gourdon and Meola, 2017; Liu et al., 2021; Miller et al., 2021). Among CNS symptoms, beside cognitive dysfunction and behavioral abnormalities, disturbances associated with sleep are very common, affecting more than 80% of patients (Laberge et al., 2013; Subramony et al., 2020). For example, excessive daytime sleepiness (EDS), namely the inability to stay awake in situations that require wakefulness, is a symptom with a very high prevalence that seriously affects the quality of life, is often premorbid and associated with fatigue. It is rarely accompanied by changes in nocturnal EEG recordings, it is unrelated to the duration or quality of night sleep and is of central origin. It is thus considered as a primary hypersomnia in DM1 that is efficaciously treated with the psychostimulant modafinil, an effective and well-tolerated drug acting primarily on blocking catecholamines re-uptake as well as affecting serotonin, glutamate, gamma amino-butyric acid, orexin, and histamine systems in the brain (Talbot et al., 2003; Minzenberg and Carter, 2008; Laberge et al., 2013; Subramony et al., 2020).

Sleep-related disorders are assessed in humans initially with diaries, questionnaires and self-reports and ultimately and more objectively with tests measuring the latency to fall asleep or maintain wakefulness during the day or using the gold standard polysomnographic methods (combining video, EEG, EMG etc.). The disadvantage of these highly recommended tests is that they have to take place in laboratory settings with the risk of influencing a measure that is greatly affected by environmental inputs (Baiardi and Mondini, 2020). This inconvenience can be partly overcome by the use of actigraphy, an objective assessment, that unobtrusively monitors activity and wake/sleep patterns at home and during daily activities (Liguori et al., 2021). Similar technologies are also recently adopted in patients as well as in the general population and easily accessible with common smart phones, smart watches and different wearables or “nearables” (Haghi et al., 2017; Kourtis et al., 2019; Hanein and Mirelman, 2021).

A similar type of monitoring can be obtained in animal models of neurodegenerative and neuropsychiatric diseases through the use of home cage monitoring (HCM) systems (Voikar and Gaburro, 2020). These systems allow the 24/7 continuous detection of mouse activity for long periods of time by collecting spontaneous in-cage activity data both during the day and especially at night when mice are more active (Richardson, 2015; Bains et al., 2018; Pernold et al., 2019). They require minimal effort from experimenters and care takers and most importantly they improve animal welfare by having less stressful test conditions and removing potential sources of variation. In animal models, the use of non-invasive monitoring tools is crucial especially in those mutant strains that are particularly frail due to their pathological phenotype.

In a previous study (Golini et al., 2020) we could follow the entire progression of the disease (from asymptomatic to humane endpoint stage) in an amyotrophic lateral sclerosis mouse model monitoring the home cage activity of SOD1G93A mice for more than 4 months using the Digital Ventilated Cage (DVC^®^) system (Iannello, 2019). Analyzing the pattern of activity, we were able to develop a new digital biomarker called Regularity Disruption Index (RDI) to potentially detect rest disturbances, and we showed that rest was significantly disrupted in SOD1G93A mice at the symptomatic stage concurrently with other neuromuscular symptoms.

For this study we aimed to verify if, in DMSXL mice, an established model of DM1, we could detect rest disturbances that would mimic some aspects of the human sleep-related symptomatology. DMSXL mice exhibit a pathologic neuromuscular phenotype and CNS-related abnormalities similar to those observed in patients (Huguet et al., 2012; Hernández-Hernández et al., 2013; Sicot et al., 2017; De Serres-Bérard et al., 2021).

We found that DMSXL mice rest longer than wild-types when they are in their active phase (dark period) and this novel phenotype could model one of the most prominent DM1 CNS-related symptom, namely excessive daytime sleepiness (EDS). Moreover, the home cage monitoring technology proved to be the most appropriate to detect this type of sleep-related phenotype without disturbing the animals and consequently without impairing their welfare further.

## Materials and methods

### Subjects

Transgenic DMSXL mice (C57BL/6 background) carry a human genomic fragment of 45 kb of the DM1 locus with an expansion of >1,000 CTG repeats in the *DMPK* gene (Seznec et al., 2000; Gomes-Pereira et al., 2007). A mouse colony was established at the IBBC-EMMA-Infrafrontier animal facility (Monterotondo, Italy) by crossing hemizygous individuals. Progeny was genotyped by PCR of tail samples DNA with primers specific to identify the transgenic status of mutant mice as well as WT individuals (González-Barriga et al., 2021), and sex matched litters were group housed in standard IVC cages (Tecniplast S.p.A., Buguggiate, Italy) with food and chlorinated, filtered water *ad libitum*. Room temperature was 21 ± 2^°^C, relative humidity was 50–60%, and mice were kept in a 12 h light/dark cycle with lights on from 07:00 a.m. to 07:00 p.m.

Animals were subjected to an experimental protocol approved by the Veterinary Department of the Italian Ministry of Health (No. 832/2019-PR), and experiments were conducted according to the ethical and safety rules and guidelines for the use of animals in biomedical research provided by the relevant Italian laws and European Union’s directives (No. 86/609/EEC and subsequent).

### Welfare issues

DMSXL homozygous mice need special care, particularly during the first month after birth. As described by Huguet et al. (2012), they showed high perinatal mortality (around 50%) and were severely underweight (Supplementary Figure 1). To cope with these issues, we fed all mice with an energy rich diet both for breeding and weaned litters (EMMA23, Mucedola, Settimo Milanese, Italy). Furthermore, gel diet (DietGel76A; Clear H20, Westbrook, ME, USA) and wet food were provided on the cage floor. Also, some homozygous mice displayed misalignment of the upper incisors that had to be trimmed regularly for feeding properly. The welfare issues described continued for the entire duration of the study.

### Experimental design

Home cage monitoring experiments have been performed using male and female homozygous DMSXL mice and their wild-type (WT) littermates coming from four different runs/cohorts of independent litters born from January to October 2021. Mice were housed two per cage of the same sex and genotype in Digital Ventilated Cages (DVC^®^) from the age of 7 until 12 weeks. In this 5 weeks study mouse activity was automatically measured 24/7. As the home cage is the experimental unit, when a subject died prematurely or had to be sacrificed for showing a reduction of >25% of initial body weight (humane endpoint), we had to exclude the whole cage from the experiment. At the end, the experimental groups were as follows: (1) Males, WT *n* = 20 (10 cages); (2) Males, DMSXL *n* = 20 (10 cages); (3) Females, WT *n* = 22 (11 cages); (4) Females, DMSXL *n* = 16 (eight cages). All mice were weighed weekly at the same time of cage change, while every 2 or 3 days homozygous DMSXL mice were observed for general health and teeth condition. Diet gel or wet food provided on the cage floor didn’t affect the DVC^®^ activity measurements.

### Home cage monitoring: DVC^®^ activity metrics

The Digital Ventilated Cage (DVC^®^) rack is a home-cage monitoring system, where an electronic board consisting of 12 capacitance sensing electrodes is positioned below each cage. To capture the activity of the mice inside the cage, we used the Animal Locomotion Index Smoothed (DVC^®^ Analytics, Tecniplast S.p.A., Buguggiate, Italy), which is based on activation density (Iannello, 2019). This metric considers that an electrode is activated by the animals when the difference between two consecutive capacitance measurements is larger than a fixed threshold (λ), which is conveniently chosen to separate noise induced capacitance variations from animal movements (Iannello, 2019).

We used the Regularity Disruption Index (RDI) to quantify the regularity of the pattern of activity of the animals during light and dark phases (Golini et al., 2020). RDI is determined by computing sample entropy on the minute activity time series (both day and night time), to which is previously applied a Butterworth band-pass filter (see Golini et al., 2020). RDI approaching 0 indicates that the activity time series consists of very similar minute activity levels (comparable to rest behavior), whereas a very fragmented time series, in which all minutes are very different with each other, gives a high RDI (comparable to non-rest behavior).

To further study the rest behavior of the animals, we firstly determined the least active consecutive hour, which may correspond to a consistent period of rest and is usually located during the lights-on phase (Golini et al., 2020), by selecting the 60 consecutive minutes with the lowest average activity across the day (24 h). We then considered non-activity, corresponding to both mice being immobile, as a measure of rest. For this purpose, we used the lowest possible threshold (λ) to consider an electrode being activated, in order to better discriminate between complete immobility and movement of the animals, even if very small or slow (λ = 1.25 to compare two consecutive 1 s average windows; Iannello, 2019). We then calculated the percentage of time of 0 activity over the 24 h of the day. Finally, we determined the rest bouts as sequences of consecutive 0 activity of a minimum length of 40 s [according to Pack et al. (2007)] and divided them in three bins of different durations (<5 min, 5–15, >15 min). The amount of rest of each bin was time-weighted and normalized by the total amount of rest during light and dark phases, and then used to produce two time-weighted frequency histograms [similarly to Mochizuki et al. (2004)].

### Statistical analysis

On most datasets, we performed a non-parametric repeated measures statistical test, by using the nparLD rank-based analysis of variance-type statistic (Noguchi et al., 2012), with Sex and Genotype as whole-plot (between-subjects) factors and Week as sub-plot (within-subjects) factor. We ran post-hoc analysis where possible, using nparLD or two-sample Wilcoxon Tests, Bonferroni corrected. We used Python to process and visualize data and R to run all the statistics, with significance level α = 0.05. We excluded days of cage change from the analysis.

## Results

**FIGURE 1:**
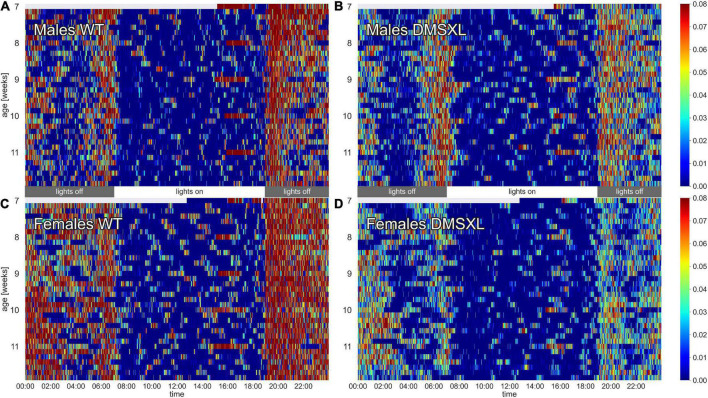
Heatmaps of activity. The figure depicts average minute activity across 24 h and over 5 weeks in four representative cages of wild-type (WT) male **(A)**, DMSXL male **(B)**, WT female **(C)**, and DMSXL female **(D)** mice (*n* = 2 per cage). Lights-on is from 7 a.m. to 7 p.m.

The activity of the animals was monitored by the DVC^®^ system 24/7 for 5 weeks. Heatmaps of four representative cages of male and female wild-type (WT) and DMSXL mice show the activity distribution across the 24 h of the day and across the 5 weeks of the experiment (Figure 1). As expected, the highest activity was recorded during night time, which corresponds to the active phase of the mice (Pernold et al., 2019), and especially across the times of light/dark transitions (Fuochi et al., 2021; Supplementary Figure 2). Activity in cages with WT mice was clearly higher than DMSXL mice throughout the whole period and especially during the lights-off phase. As expected, females were more active than males in WT cages (Pernold et al., 2019), but notably not in DMSXL mice. Activity seemed to be stable over all the 5 weeks of the experiment, which corresponded to 7–12 weeks of age (Supplementary Figure 3). Interestingly, the heatmaps show longer periods of inactivity (color-coded in dark blue) during the lights-off phase in cages with DMSXL mice compared to cages with WT.

### Activity and RDI during light and dark phase

**FIGURE 2:**
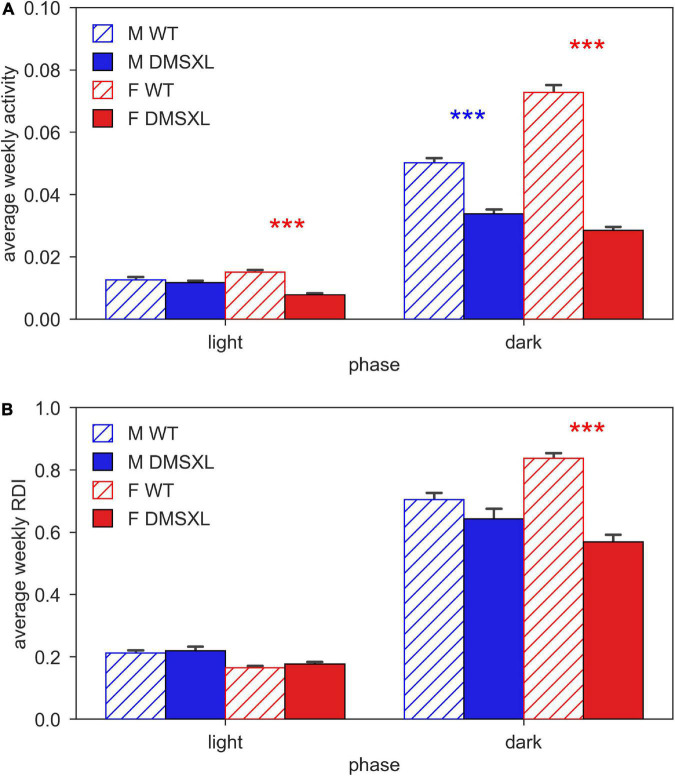
Activity and regularity disruption index (RDI) during light and dark phase. Panel **(A)** shows the average weekly activity (±SEM) and panel **(B)** shows the average weekly RDI (±SEM), both over light and dark phases across the 5 weeks of experiment in male (M, blue) and female (F, red) cages of wild-type (WT) (diagonal lines) and DMSXL (filled bar) mice. N of cages per group: M WT = 10; M DMSXL = 10; F WT = 11; F DMSXL = 8. Bonferroni-corrected *post-hoc* tests to compare DMSXL and WT males (blue asterisks), and DMSXL and WT females (red asterisks), (***p < 0.001 nparLD test with genotype and week factors).

We firstly computed the average activity of cages of WT and DMSXL, male and female mice during day and night time (Figure 2A). As expected, activity was much higher during the lights-off phase, and in WT females compared to males. We observed that DMSXL mice were significantly less active compared to WT, both during light (nparLD test, Genotype: Statistic = 8.647, df = 1, *p* < 0.01) and dark period (nparLD test, Genotype: Statistic = 127.148, df = 1, *p* < 0.001). This difference was more remarkable in females (light: nparLD test, Genotype × Sex interaction: Statistic = 8.231, df = 1, *p* < 0.01; dark: nparLD test, Genotype × Sex interaction: Statistic = 16.996, df = 1, *p* < 0.001).

We computed the Regularity Disruption Index (RDI) to capture the fragmentation of the activity pattern during the 12 h of day and night time (Figure 2B). As expected, RDI was much higher in all the groups during the lights-off phase, but with DMSXL mice showing a less fragmented activity than WT mice (nparLD test, Genotype: Statistic = 18.014, df = 1, *p* < 0.001), with the greatest difference displayed in females (nparLD test, Genotype × Sex interaction: Statistic = 9.598, df = 1, *p* < 0.05). A less fragmented activity pattern likely indicates more rest (Golini et al., 2020).

### Rest distribution

**FIGURE 3:**
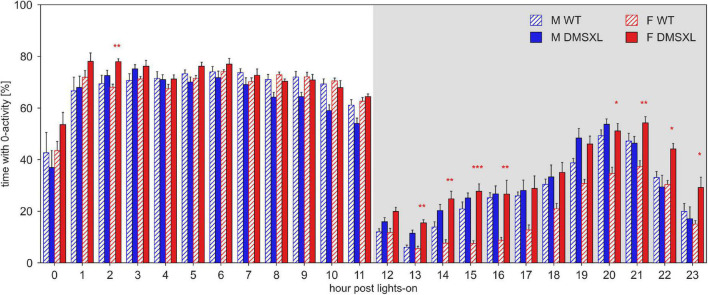
Percentage of time with 0 activity across the 24 h of the day. Average percentage of time (±SEM) per hour post lights-on, across the 5 weeks of experiment in male (M, blue) and female (F, red) cages of wild-type (WT) (diagonal lines), and DMSXL (filled bar) mice. N of cages per group: M WT = 10; M DMSXL = 10; F WT = 11; F DMSXL = 8. Bonferroni-corrected *post-hoc* tests to compare DMSXL and WT males, and DMSXL and WT females (red asterisks), per each hour (*p < 0.05, **p < 0.01, ***p < 0.001 two sample Wilcoxon test).

To investigate the distribution of the rest pattern during the day, we computed the percentage of 0-activity per each of the 24 h of the light/dark pattern (Figure 3). As expected, the higher percentage of inactivity was found during the light hours (nparLD test, Hour: Statistic = 311.087, df = 5.785, *p* < 0.001). DMSXL mice showed a remarkably higher percentage of rest than WT mice (nparLD test, Genotype: Statistic = 7.722, df = 1, *p* < 0.01), especially during the dark phase (nparLD test, Genotype × Hour interaction: Statistic = 4.187, df = 5.785, *p* < 0.001) and in females (nparLD test, Genotype × Sex interaction: Statistic = 8.996, df = 1, *p* < 0.01).

**FIGURE 4:**
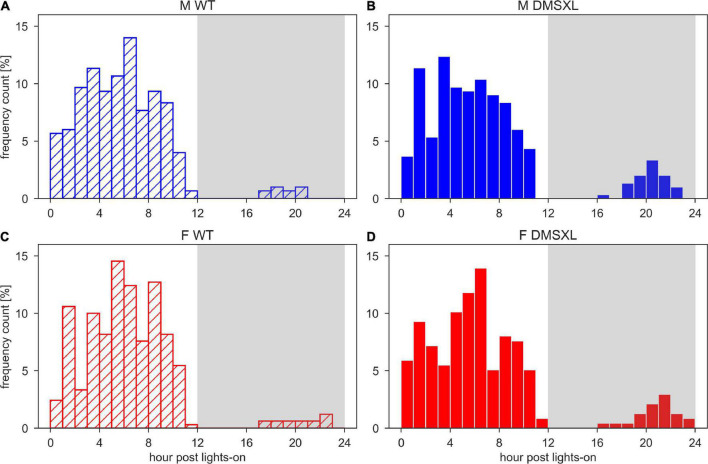
Histograms of the time of the least active hour. Time of the day of the least active hour per each cage and each day of the 5 weeks of experiment in wild-type (WT) male **(A)**, DMSXL male **(B)**, WT female **(C)**, and DMSXL female **(D)** mice. N of cages per group: M WT = 10; M DMSXL = 10; F WT = 11; F DMSXL = 8.

We also calculated the least active consecutive hour, which may correspond to a consistent period of rest and thus usually located during the lights-on phase, as shown in Figure 4. We observed that the least active hour was rarely found during the dark period, but more likely in DMSXL mice than WT: around 10% in both DMSXL males and females, and 3 and 4% in WT males and females, respectively. We then calculated the average activity within the least active hour both during the light and dark phase (Supplementary Figure 4) and we observed that it was significantly lower in DMSXL compared to WT mice (nparLD test, Genotype: Statistic = 54.618, df = 1, *p* < 0.001), and especially in females (nparLD test, Genotype × Sex interaction: Statistic = 11.483, df = 1, *p* < 0.001), only during the dark phase.

**FIGURE 5:**
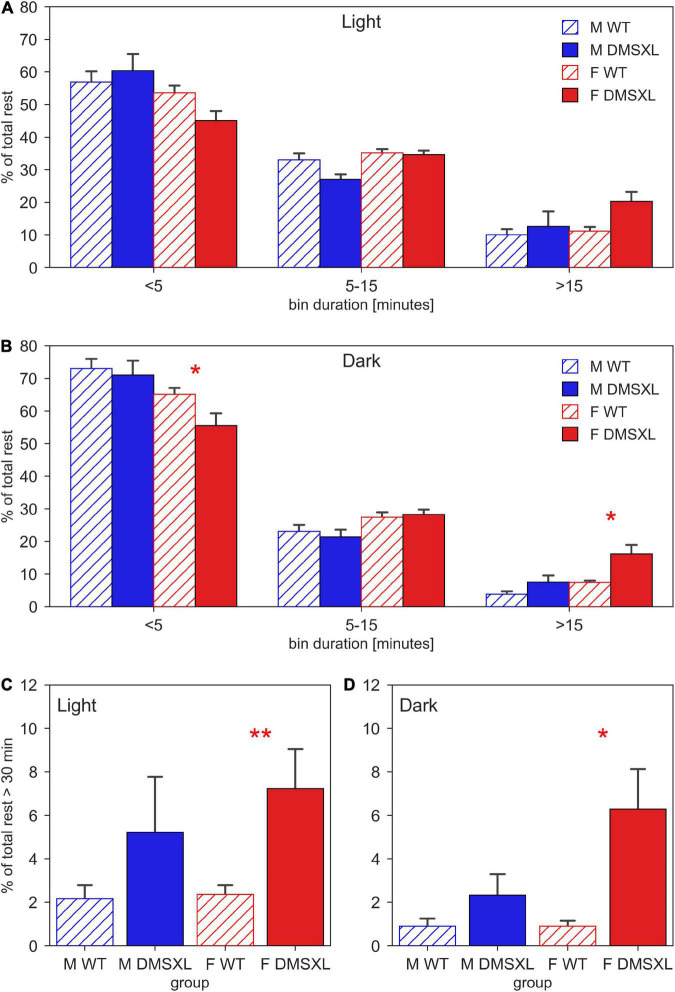
Duration of resting bouts. Time-weighted frequency histogram of the durations of rest bouts during light **(A)** and dark phases **(B)**, across the 5 weeks of experiment in male (M, blue) and female (F, red) cages of wild-type (WT) (diagonal lines) and DMSXL (filled bar) mice. N of cages per group: M WT = 10; M DMSXL = 10; F WT = 11; F DMSXL = 8. The rest bouts (sequences of consecutive 0-activity of a minimum length of 40 s) are divided in three bins of different durations: <5 min, 5–15, >15 min. The bottom panels focus on the rest bouts longer than 30 min during light **(C)** and dark **(D)** phases. In **(B–D)**, Bonferroni-corrected *post-hoc* tests were performed to compare DMSXL and WT males (blue asterisks), and DMSXL and WT females (red asterisks) per each bin duration, (*p < 0.05, **p < 0.01 two sample Wilcoxon test).

Finally, we displayed the distribution of the duration of the rest bouts, computed as sequences of consecutive 0-activity lasting more than 40 s (Figure 5). We observed that both during light (Figure 5A) and dark period (Figure 5B), the majority of rest during the 24 h of the day was performed within short bouts (<5 min), especially in males (light: nparLD test, Sex: Statistic = 9.365, df = 1, *p* < 0.01, dark: nparLD test, Sex: Statistic = 12.165, df = 1, *p* < 0.001). Very interestingly, DMSXL mice showed more rest bouts longer than 15 min than WT, especially during the dark phase (nparLD test, Genotype × Bin Duration: Statistic = 3.597, df = 1.496, *p* < 0.05) and in females (nparLD test, Sex × Bin Duration: Statistic = 10.340, df = 1.496, *p* < 0.001). We then focused on consecutive rest sequences lasting more than 30 min, and we observed a remarkably higher percentage distribution in females DMSXL mice both during light (Figure 5C) and dark period (Figure 5D).

## Discussion

Using an automated home cage monitoring system (DVC^®^), we could evaluate rest behavior in DMSXL mice non-invasively for 5 weeks from 7 until 12 weeks of age. We found that the DMSXL mice had longer periods of rest during the active phase (lights-off) compared to WT littermates and this is remarkably similar to what observed in DM1 patients affected by EDS. Sleep disturbances including EDS and fatigue have been reported in a large percentage of DM1 patients (Laberge et al., 2013; Subramony et al., 2020; Sansone et al., 2021) and, as in many other neurodegenerative diseases, they impact on the quality of life of patients more than other symptoms (Laberge et al., 2004; Heatwole et al., 2012).

Central nervous system (CNS) manifestations and most importantly fatigue and sleep disturbances as EDS are present in all forms of DM1, from congenital to adult-onset (De Serres-Bérard et al., 2021). DMSXL mice can be considered a reliable model of the most severe early-onset forms of DM1 since they present phenotypes corresponding to the typical CNS and non-CNS clinical symptoms of the disease found from birth and in childhood/juvenile forms (De Serres-Bérard et al., 2021). Specifically for CNS-related phenotypes, DMSXL mice show reduced exploration and memory deficits as well as synaptic dysfunction associated with dysregulation of pre-synaptic proteins (Hernández-Hernández et al., 2013). In addition, deficits in motor coordination due to cerebellar glial and neuronal abnormalities caused by glutamate abnormal levels have been also described in DMSXL mice (Sicot et al., 2017). Finally, cognitive and REM sleep disorders as well as increased time of immobility in the absence of neuromuscular or locomotor deficits have been found in DM1 linked *Mbnl2* knock-out mice (Charizanis et al., 2012; Edokpolor et al., 2022). Our study is the first to describe a sleep-related phenotype in DMSXL mice as a relevant mouse model of early-onset forms of DM1.

Objective assessment of sleep disturbances is not always feasible and evaluation of EDS and fatigue (a symptom often associated) usually relies on subjective reports and questionnaires (Laberge et al., 2009). Tests measuring the latency to fall asleep or maintain wakefulness during the day or even polysomnographic methods are indeed objective but imply laboratory-like settings that could not entirely correlate with sleep-associated behaviors in the home environment or during daily life activities. Similarly, in animal models, assessment of sleep using EEG, EMG and other invasive techniques can be unsuitable in specific mutants with severe welfare issues. Home cage monitoring allows the continuous and unobtrusive detection of many parameters including activity and rest (Voikar and Gaburro, 2020). While any detailed sleep analysis still requires EEG, data collected with these systems can provide behaviorally defined proxies of sleep (e.g., >40 s of immobility, Pack et al., 2007; Fisher et al., 2012; Brown et al., 2016; Bains et al., 2018) that have been highly correlated to EEG measurements and are particularly adequate for longitudinal studies (lasting weeks or even months) and with frail animals, like the DMSXL mouse model.

DMSXL mice, monitored in their home cages showed lower levels of activity compared to WT littermates and longer periods of inactivity, especially in female subjects (Figures 1, 2A). A further analysis of their activity allowed us to compute the time spent inactive (0 activity), considered as rest. We used a lower threshold than the standard activation density metric, in order to exclude any possible animal micro-movement (Iannello, 2019). Interestingly, the percentage of time resting was higher in DMSXL mice, especially during the dark period (active phase) and in females (Figure 3). Similarly, when calculating a consistent period of rest as the least active hour within 24 h (Golini et al., 2020), this was found predominantly, as expected in mice, during the lights-on period but for DMSXL mice more than in WT, it was also found during the dark period (Figure 4). Moreover, the average activity in the least active hour, especially in females and during the lights-off phase, was significantly lower in DMSXL mice than in WT (Supplementary Figure 4). Finally, we were able to determine rest bouts as sequences of consecutive 0 activity of a minimum duration of 40 s, and we found that the frequency of rest bouts longer than 15 or even 30 min was higher in DMSXL mice compared to WT, especially in females and predominantly in the dark phase (Figure 5). Inactivity lasting more than 40 s is an established measure of behaviorally defined sleep in mouse models, as already described by Pack et al. (2007), Fisher et al. (2012), Brown et al. (2016), in which different home cage monitoring activity measurements have been correlated and validated with EEG recordings. Notably, female DMSXL mice seemed to display even longer period of rest compared to males, confirmed with all DVC^®^ activity metrics. Similar sex differences have been observed in DM1 patients enrolled in a study conducted within the OPTIMISTIC trial (NCT02118779). In this trial it was verified the efficacy of cognitive behavioral therapy and graded exercise to reduce fatigue and sleepiness in both sexes (Okkersen et al., 2018; Mahon et al., 2022). In a recent study on EDS in a small cohort of adult-onset DM1 patients, a tendency to have a faster propensity to fall asleep and higher daytime sleepiness scores could be found in female subjects more than males (Sansone et al., 2021). Additionally, the importance of gender on the severity of DM1 symptoms was emphasized in Dogan et al. (2016) where again a non-significant tendency in females to show higher “somnolence” was reported. Unfortunately, studies in mouse models of DM1 rarely test and evaluate sex differences, our study could thus contribute to the understanding of gender as an influencing factor in the manifestations of this pathology.

DMSXL female mice also showed a significantly reduced RDI in the dark phase compared to WT, indicating a more stable pattern of activity, thus probably correlated to more and longer periods of rest (Figure 2B). RDI (Regularity Disruption Index) is another metric that we proposed as a reliable measure of rest disturbance in Golini et al. (2020) where we applied it to uncover sleep-related abnormalities in a mouse model of ALS. Notably, RDI in SOD1G93A mice (Golini et al., 2020) has been considered a very reliable digital biomarker as reported in a recent systematic review on digital biomarkers important to ameliorate early diagnosis and more tailored treatments for neuromuscular diseases including ALS and muscular dystrophies (Youn et al., 2021).

In conclusion, we demonstrated for the first time in the DMSXL mouse model the occurrence of a phenotype related to symptoms similar to what observed in DM1 patients, namely sleep dysfunctions, and in particular EDS. The use of a non-invasive method to screen for sleep-related behaviors in a more familiar environment is especially recommended in mutant strains displaying conspicuous welfare issues. The possibility of long-term monitoring of the parameters described as well as other health-related measures can be of help to better evaluate the efficacy of treatments over time.

## Data availability statement

The raw data supporting the conclusions of this article will be made available by the authors, without undue reservation.

## Ethics statement

The animal study was reviewed and approved by the Veterinary Department of the Italian Ministry of Health (No. 832/2019-PR), and experiments were conducted according to the ethical and safety rules and guidelines for the use of animals in biomedical research provided by the relevant Italian laws and European Union’s directives (no. 86/609/EEC and subsequent).

## Author contributions

MRi, SM, and EG: conceptualization, validation, visualization, methodology, writing—original draft, and review and editing. MRi: data handling, formal analysis, and software. EG and SM: investigation. FS, MRa, and GG: resources. MRi, SM, and GF: supervision. All authors contributed to the article and approved the submitted version.
