# Supplementary material for: Excessive rest time during active phase is reliably detected in a mouse model of myotonic dystrophy type 1 using home cage monitoring
Source: Front Behav Neurosci. 2023 Mar 2;17:1130055. doi: 10.3389/fnbeh.2023.1130055 (PMC10017452; doi:10.3389/fnbeh.2023.1130055)
Supplement: Supplementary file 1 [file Presentation_1.pdf]

Excessive rest time during active phase is reliably detected in a mouse model of Myotonic Dystrophy type 1 using home cage monitoring

Supplementary Material

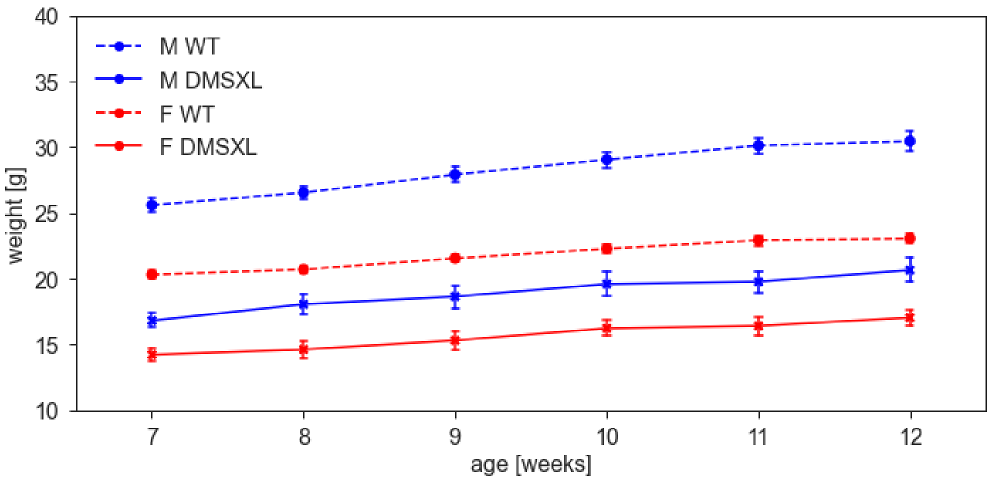

Supplementary Figure 1. Body weights. Average body weight (±SEM) of the animals, across the five weeks of the experiment.

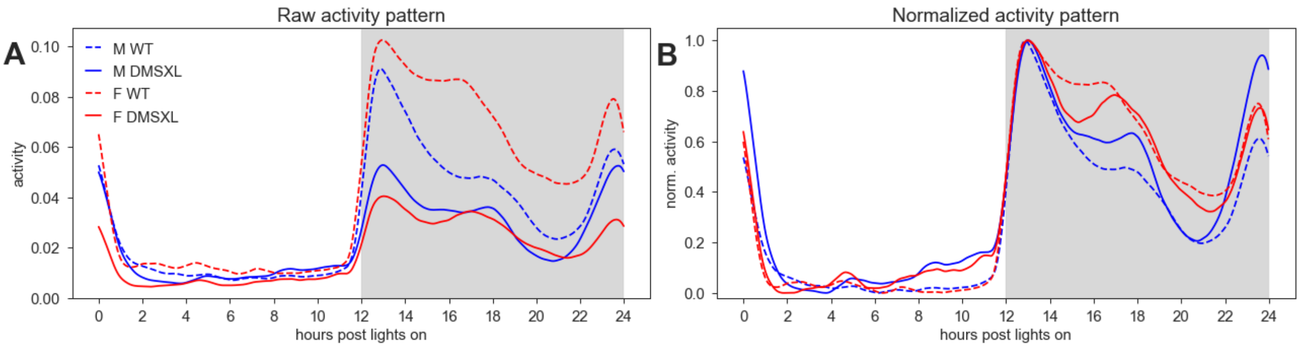

Supplementary Figure 2. Activity pattern over 24 h. In panel A, we considered the minute activity time series (1440 minutes), smoothed with a 1-hour moving average. In panel B, we normalized each of the smoothed 1440-minutes time series to its peak activity so that the maximum value is one. We then averaged the curves across all days and cages within the same group. In A, we can observe the average absolute activity across the 24 hours of the day, while in B we can better appreciate the shape of the circadian pattern, regardless of the total amount of activity.

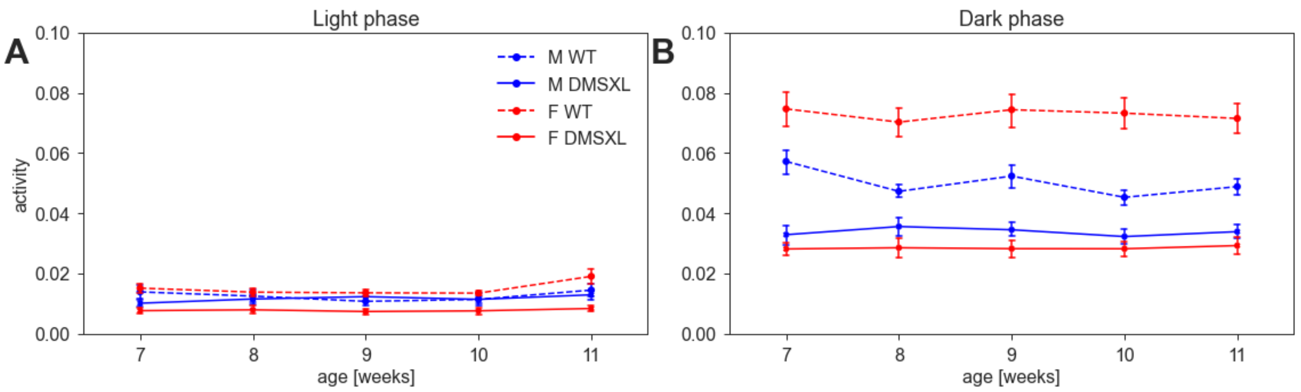

Supplementary Figure 3. Average weekly activity. Average activity (±SEM) during light (panel A) and dark phases (panel B), across the five weeks of the experiment (from age 7 to 12 weeks).

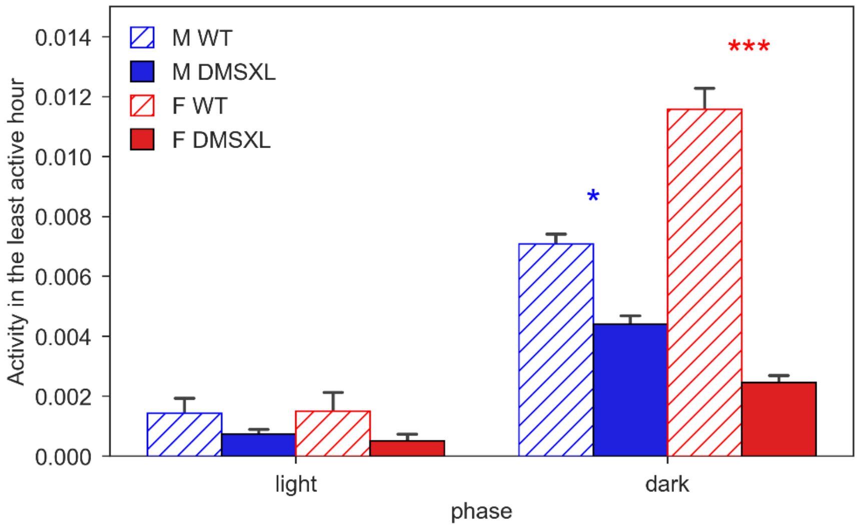

Supplementary Figure 4. Activity within the least active hour. Average weekly activity (±SEM) within the least active hour computed within both light and dark phase, across the 5 weeks of experiment in male and female cages of WT and DMSXL mice. Bonferroni-corrected post-hoc tests to compare DMSXL and WT males (blue asterisks), and DMSXL and WT females (red asterisks), (*p<0.05, **p < 0.01, ***p < 0.001 nparLD test with strain and week factors).
